# Curcumin improves glycolipid metabolism through regulating peroxisome proliferator activated receptor γ signalling pathway in high-fat diet-induced obese mice and 3T3-L1 adipocytes

**DOI:** 10.1098/rsos.170917

**Published:** 2017-11-15

**Authors:** Yanyun Pan, Dandan Zhao, Na Yu, Tian An, Jianan Miao, Fangfang Mo, Yujie Gu, Dongwei Zhang, Sihua Gao, Guangjian Jiang

**Affiliations:** 1 Traditional Chinese Medicine School, Beijing University of Chinese Medicine, Beijing 100029, People's Republic of China; 2 Diabetes Research Center, Beijing University of Chinese Medicine, Beijing 100029, People's Republic of China

**Keywords:** curcumin, glycolipid metabolism, obese mice, 3T3-L1 adipocytes, peroxisome proliferator activated receptor γ

## Abstract

Curcumin is an active component derived from *Curcuma longa* L. which is a traditional Chinese medicine that is widely used for treating metabolic diseases through regulating different molecular pathways. Here, in this study, we aimed to comprehensively investigate the effects of curcumin on glycolipid metabolism *in vivo* and *in vitro* and then determine the underlying mechanism. Male C57BL/6 J obese mice and 3T3-L1 adipocytes were used for *in vivo* and *in vitro* study, respectively. Our results demonstrated that treatment with curcumin for eight weeks decreased body weight, fat mass and serum lipid profiles. Meanwhile, it lowered fasting blood glucose and increased the insulin sensitivity in high-fat diet-induced obese mice. In addition, curcumin stimulated lipolysis and improved glycolipid metabolism through upregulating the expressions of adipose triglyceride lipase and hormone-sensitive lipase, peroxisome proliferator activated receptor γ/α (PPARγ/α) and CCAAT/enhancer binding proteinα (C/EBPα) in adipose tissue of the mice. In differentiated 3T3-L1 cells, curcumin reduced glycerol release and increased glucose uptake via upregulating PPARγ and C/EBPα. We concluded that curcumin has the potential to improve glycolipid metabolism disorders caused by obesity through regulating PPARγ signalling pathway.

## 1. Introduction

Obesity has become one of the most challenging health problems in the twenty-first century. The latest statistics showed that more than 1.0 billion adults worldwide were overweight; of these, over 400 million were obese [1]. It is usually accompanied with complex metabolic disorders, and significantly increases the risk of several common and serious diseases such as diabetes mellitus, hyperlipidaemia, hypertension, cardiovascular disease and non-alcoholic fatty liver disease [2]. Obesity is characterized by excessive accumulation of body fat (triglycerides (TGs)) in adipose tissue [3]. Interestingly, adipose tissue is not only an energy storage depot, but also a well-known endocrine organ secreting several cytokines and adipokines that are key determinants of whole-body energy homeostasis [4,5]. Furthermore, dysfunction or excessive adipose storage of adipose tissue may lead to ectopic lipid accumulation and lipotoxicity in obese individuals and block insulin signalling transport [6,7].

**Figure 1.**
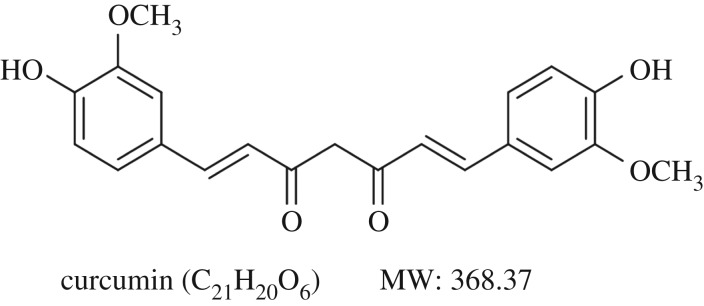
Chemical structure of curcumin. Its molecular formula is C_21_H_20_O_6_. MW (molecular weight): 368.37.

Currently, various drugs have been approved by the US Food and Drug Administration to treat obesity in adults, but serious side effects of these drugs have limited their application [8,9]. Fortunately, several commonly used medicinal plants and their bioactive compounds have been shown to prevent obesity and its related metabolic disorders in traditional Chinese medicine. Among them, curcumin (1,7-bis(4-hydroxy-3-methoxyphenyl)-1,6-heptadiene-3,5-dione, figure 1) is an active component derived from traditional herbal remedy and dietary spice turmeric (*Curcuma longa* L.), widely used in Ayurvedic and Chinese medicine for thousands of years [10,11]. A large amount of experiments have demonstrated various biological activities of curcumin, including anti-inflammatory, anti-cancer and antioxidant [12,13]. Curcumin protects rats from chronic inflammation observed in metabolic syndrome through inhibition of activation of NF-κB signalling [14,15]. In addition, curcumin suppresses oxidation response of the pancreatic β cells by targeting PI3-k/Akt-mediated pathway and improves insulin-stimulated protein kinase B phosphorylation in the liver [16]. These reports indicated that curcumin exerted protective effects on metabolic diseases. Emerging evidence demonstrated that peroxisome proliferator activated receptor γ (PPARγ) is mainly involved in regulating lipid metabolism, insulin sensitivity and glucose homeostasis [17]. However, little is reported about the effects of curcumin on glycolipid metabolism through regulating PPARγ signalling both *in vivo* and *in vitro*.

Here, we investigated the effect of curcumin on glycolipid metabolism in high-fat diet (HFD)-induced obese mice and 3T3-L1 adipocytes and the potential mechanisms were also explored.

## 2. Material and methods

### 2.1. Chemicals

Curcumin was purchased from Sigma Chemical Co. (the purity was 99% tested by HPLC analysis). Blood glucose and TG kits were obtained from Beijing Leadman Biochemical Co. Ltd (Beijing, China). Assay kits for total cholesterol (TC), low density lipoprotein cholesterol (LDL-C) and high density lipoprotein cholesterol (HDL-C) were obtained from Zhongsheng Beikong Biotechnology and Science Inc. (Beijing, China). Dulbecco's modified Eagle's medium (DMEM), penicillin–streptomycin (P/S), phosphate-buffered saline and trypsin--EDTA were purchased from Gibco (Grand Island, NA, USA). Insulin, dexamethasone (DEX), 3-isobutyl-1-methylxanthine (IBMX) and Oil Red O were purchased from Sigma (St Louis, MO, USA).

### 2.2. Animals

Male C57BL/6 J mice (17–20 g, six weeks of age) were purchased from Beijing HFK Bioscience Co. Ltd (Beijing, China). The mice were housed in the clean level condition animal housing facilities (permit number SCXK (Jing) 2016-0038), temperature of 22 ± 2°C, humidity of 55 ± 5% and 12 L : 12 D cycle with free access to chow and water. All protocols were approved by the Ethics Committee of Beijing University of Chinese Medicine.

### 2.3. Experiment in mice

Mice were divided into two groups to receive different diets. Those in the control group were given AIN-96G diet with 5% corn oil (ND, *n* = 10), and the rest were fed with an MD12302 HFD containing 45% fat. The mice in the HFD group were confirmed as having obesity if their body weight was 20% greater than the mean of the control group. Then curcumin (HFD + Cur, *n* = 10) or saline (HFD, *n* = 10) were given by oral gavage for additional eight weeks. Body weight and fasting blood glucose (FBG) level were monitored weekly. The fat mass and oral glucose tolerance test (OGTT) were conducted at weeks 4 and 8. After eight-week treatment, mice were euthanatized with 1% sodium pentobarbital (0.4 ml/100 g) after fasting for 12 h. Subsequently, blood was collected from the abdominal aorta and centrifuged for 15 min at 3000 r.p.m. to obtain the serum. Epididymal white adipose tissue (WAT) was harvested from the mice. All samples for real-time polymerase chain reaction (RT-PCR) and western blotting analysis were stored at −80°C.

### 2.4. Oral glucose tolerance test

An OGTT was conducted at weeks 4 and 8. After fasting overnight, the oral glucose load was then administered at a dose of 2 g kg^−1^ body weight. Then, glucose contents were measured from the tail vein at 0, 30, 60 and 120 min after glucose administration. Area under the curve (AUC) was used to evaluate the glucose tolerance. Calculation formula was as follows: AUC (mmol h l^−1^) = 0.5 × (BG 0 min + BG 30 min)/2 + 0.5 × (BG 30 min + BG 60 min)/2 + 1 × (BG 60 min + BG 120 min)/2.

### 2.5. Serum biochemical analysis

Serum TG, TC, LDL-C and HDL-C were determined using commercial enzyme-linked immunosorbent assay kits in accordance with the manufacturer's protocols. Serum free fatty acid (FFA) level was measured using an enzymatic assay.

### 2.6. Cell culture and differentiation

The 3T3-L1 preadipocytes were cultured in DMEM supplemented with 10% fetal bovine serum and 1% P/S at 37°C, 5% CO_2_ saturated humidity. The 3T3-L1 preadipocytes were grown to confluence in 24-well culture plates and differentiation was induced using a cocktail method. Briefly, cells were incubated in DMEM supplemented with MDI (0.5 mM IBMX, 1.0 µM DEX and 10 µg ml^−1^ insulin) for 2 days. Then the media were replaced with DMEM containing insulin (10 µg ml^−1^) for another 2 days. Thereafter, DMEM media were renewed every 2 days until cells reached the fully differentiated phenotype. Differentiated state of 3T3-L1 cells was determined with the lipophilic dye Oil Red O. Red staining reveals lipid droplets in the cytoplasm, indicating adipocyte differentiation. Differentiated mature 3T3-L1 adipocytes were used for this study.

### 2.7. Glucose uptake and glycerol release in 3T3-L1 adipocytes

Differentiated 3T3-L1 adipocytes were incubated in DMEM without or with curcumin (0, 10, 20, 35 µM) for 48 h. The glucose uptake and glycerol release were measured using Glucose Oxidase kit and Enzychrom Glycerol Assay kit according to the manufacturer's protocols.

### 2.8. RNA isolation and real-time polymerase chain reaction

Total RNA was isolated from the WAT and 3T3-L1 adipocytes using Trizol reagent according to the manufacturer's instructions. Then RT-PCR was performed according to the manufacturer's protocol (ABI 7500, Agilent, USA). The primers used in the experiments are shown in table 1. ARBP was employed as an endogenous control for normalization. RT-PCR data were analysed using the 2^−ΔΔCT^ relative quantification method.

**Table 1.** Primer sequences used for RT-PCR.

gene	forward (5′–3′)	reverse (5′–3′)
ATGL	GTGAAGCAGGTGCCAACATTATTG	AAACACGAGTCAGGGAGATGCC
HSL	TCATGGCTCAACTCCTTCCT	GCTGCCTCAGACACATGTAG
C/EBPα	AGCCAAATCAGGGACTGCTA	GAGGGAGGTGACAGATGAGG
PPARα	CAACCCGCCTTTTGTCATAC	CAGTGGAAGAATCGGACCTC
PPARγ	GCACATGGTTCAGAGTGGAA	TATCGTCAGCCCAGCCTAAA
ARBP	TTTGGGCATCACCACGAAAA	GGACACCCTCCAGAATTTTC

### 2.9. Western blotting analysis

WAT and 3T3-L1 adipocytes were lysed in RIPA buffer with protease inhibitor cocktail and quantified using the Micro BCA Protein Assay Kit. Forty micrograms of total protein per well were separated by sodium dodecylsulfate–polyacrylamide gel electrophoresis. The protein was then transferred to PVDF membranes, and the membranes were then incubated overnight at 4°C with antibodies of adipose triglyceride lipase (ATGL), hormone-sensitive lipase (HSL), CCAAT/enhancer binding proteinα (C/EBPα), PPARγ and PPARα (1 : 1000 dilution). Secondary goat anti-rabbit immunoglobulin G antibody was incubated at a dilution of 1 : 2000. And in the end, blots were developed using ECL kit according to the manufacturer's protocol. For *in vitro* experiment, the membranes were then incubated with antibodies of C/EBPα, PPARγ (1 : 1000 dilution). Protein bands were visualized using ImageJ software. β-Actin (1 : 2000 dilution) was used as the internal control.

### 2.10. Statistical analysis

The results are presented as the mean ± standard error. One-way analysis of variance was used for multiple group comparisons, followed by Dunnett's test for comparison between two groups. The level of statistical significance was set at *p* < 0.05.

## 3. Results

### 3.1. Effects of curcumin on body weight and fat mass in high-fat diet-induced obese mice

**Figure 2.**
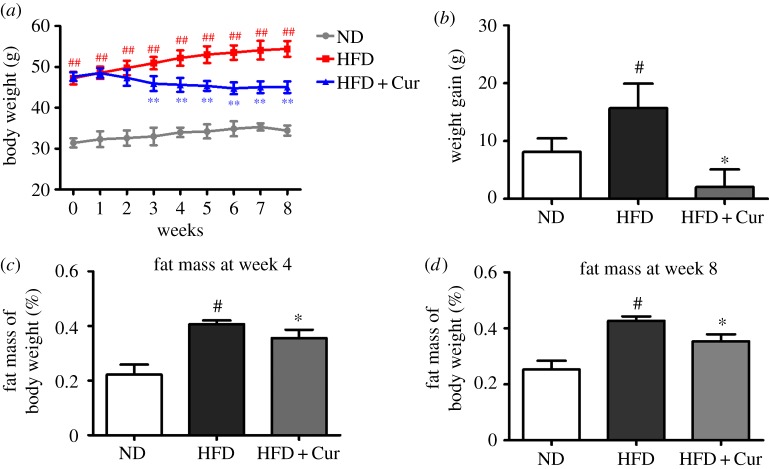
Curcumin decreased the body weight and fat mass in HFD-induced obese mice. (*a*) Body weight and (*b*) body weight gain of the mice in the ND, HFD and HFD + Cur groups; 10 samples per group were taken for each assay. The fat mass of body weight in each group at week 4 (*c*) and week 8 (*d*). Data are presented as mean ± s.e.m. in each group. Significant difference: ^#^*p* < 0.05, ^##^*p* < 0.01 versus the ND group, *p < 0.05, **p < 0.01 versus the HFD group.

As shown in figure 2*a*, the body weight of the mice in HFD significantly increased compared with mice fed in the ND group (*p* < 0.01), but curcumin (50 mg kg^−1^ d^−1^) supplement markedly reduced body weight starting from the third week. The body weight gain in the HFD group reached 15.70 g. Whereas the weight gain in the HFD + Cur group was 2.07 g and exhibited significant difference compared with the HFD group (*p* < 0.05) (figure 2*b*; electronic supplementary material, data S1). In addition, the fat mass of the mice in the HFD and HFD + Cur group at week 4 reached 0.41 ± 0.01 and 0.35 ± 0.03, respectively (figure 2*c*; electronic supplementary material, data S2). And the fat mass grew to 0.43 ± 0.02 and 0.35 ± 0.02 at week 8, respectively (figure 2*d*). These results indicated that HFD effectively induced obesity and curcumin could reduce body weight and fat mass in HFD-induced obese mice.

### 3.2. Effects of curcumin on fasting blood glucose level and oral glucose tolerance test in high-fat diet-induced obese mice

**Figure 3.**
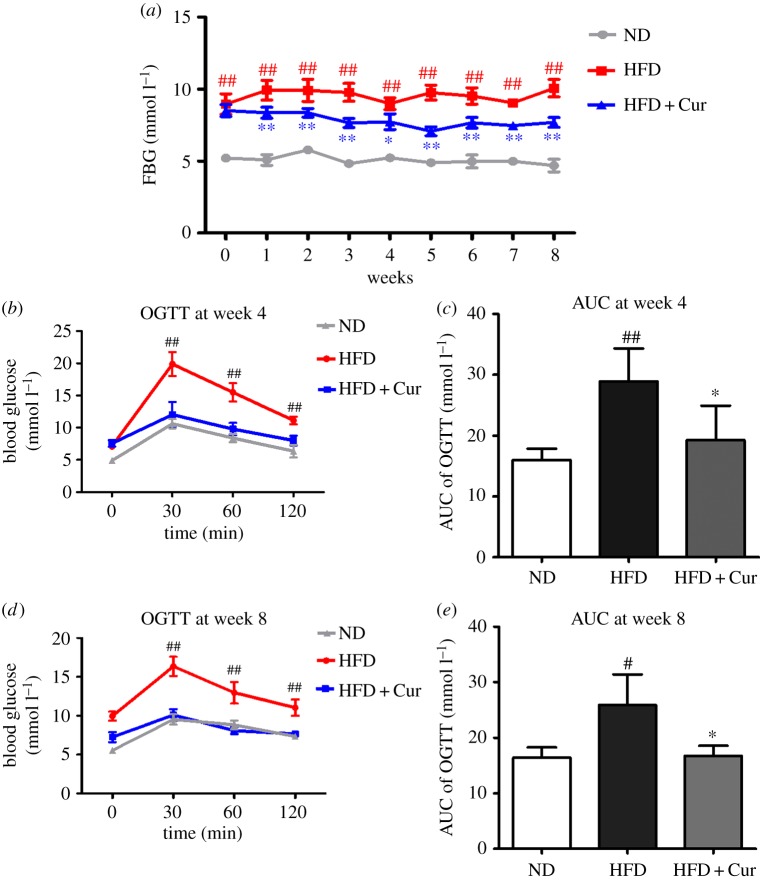
Curcumin reduced the FBG and OGTT in HFD obese mice. (*a*) The FBG level in the ND, HFD and HFD + Cur groups; 10 samples per group were taken for each assay. (*b*,*d*) The results of OGTT at week 4 and week 8. (*c*,*e*) The AUC of the OGTT at week 4 and week 8. Data are presented as mean ± s.e.m. in each group. Significant difference: ^#^*p* < 0.05, ^##^*p* < 0.01 versus the ND group, *p < 0.05, **p < 0.01 versus the HFD group.

The FBG levels of each group were measured weekly, as shown in figure 3*a*. Mice in the ND group maintained at normal FBG level, but the FBG level of the HFD group was significantly increased (*p* < 0.01). Compared with the HFD group, the FBG level began to decrease at the second week, and exhibited a maximum reduction at the fifth week in the HFD + Cur group (figure 3*a*; electronic supplementary material, data S3).

OGTT was conducted at weeks 4 and 8 (electronic supplementary material, data S4), and the results are shown in figure 3*b*–*e* . The AUC were higher in the HFD group than in the ND group. After four- and eight-week treatment with curcumin, the AUC in the HFD + Cur group were significantly reduced 33.6% and 35.5%, respectively, compared with the HFD group. In view of these results, curcumin could efficiently reduce FBG and improve obesity-induced OGTT and insulin sensitivity.

### 3.3. Effects of curcumin on serum lipid profile and free fatty acid levels in high-fat diet-induced obese mice

**Figure 4.**
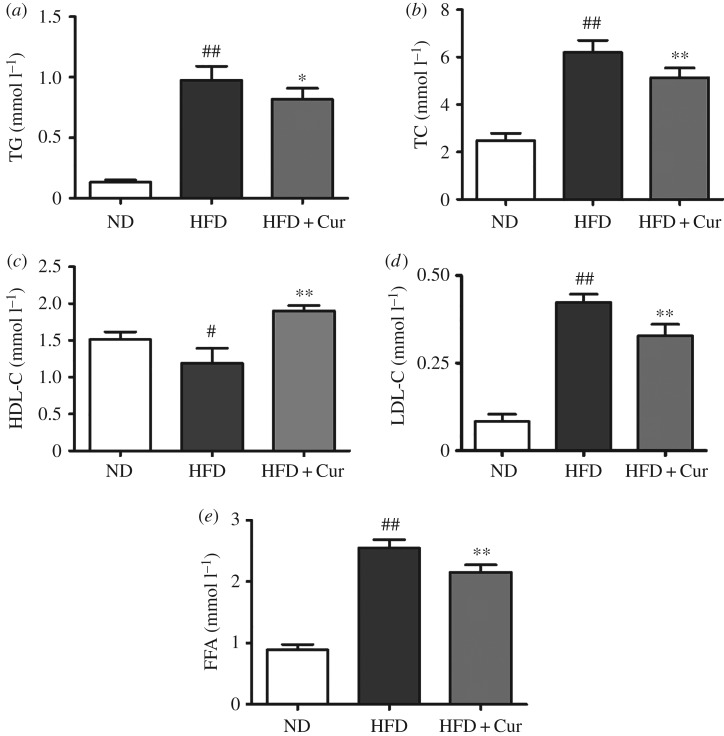
Effects of curcumin on lipid profiles and FFA level. (*a*) TG, (*b*) TC, (*c*) HDL-C, (*d*) LDL-C and (*e*) FFA in the ND, HFD and HFD + Cur groups; 10 samples per group were taken for each assay. Data are presented as mean ± s.e.m. in each group. Significant difference: ^#^*p* < 0.05, ^##^*p* < 0.01 versus the ND group, *p < 0.05, **p < 0.01 versus the HFD group.

The serum TG, TC, LDL-C and FFA level in HFD mice were markedly increased by 6.4-, 1.5-, 4.0- and 1.9-fold, respectively, compared with the ND group. However, the HDL-C level was decreased by 21.2% in HFD mice. Treatment with curcumin significantly reduced TG, TC, LDL-C and FFA level by 16.1%, 17.1%, 22.6% and 15.4%, respectively, compared with the HFD group. But the HDL-C content was decreased by 60% compared with HFD mice (figure 4; electronic supplementary material, data S5). These results demonstrated that curcumin markedly reduced the serum lipid levels and improved the disorders of lipid metabolism.

### 3.4. Effects of curcumin on mRNA and protein expression associated with peroxisome proliferator activated receptor γ signalling pathway in adipose tissue

**Figure 5.**
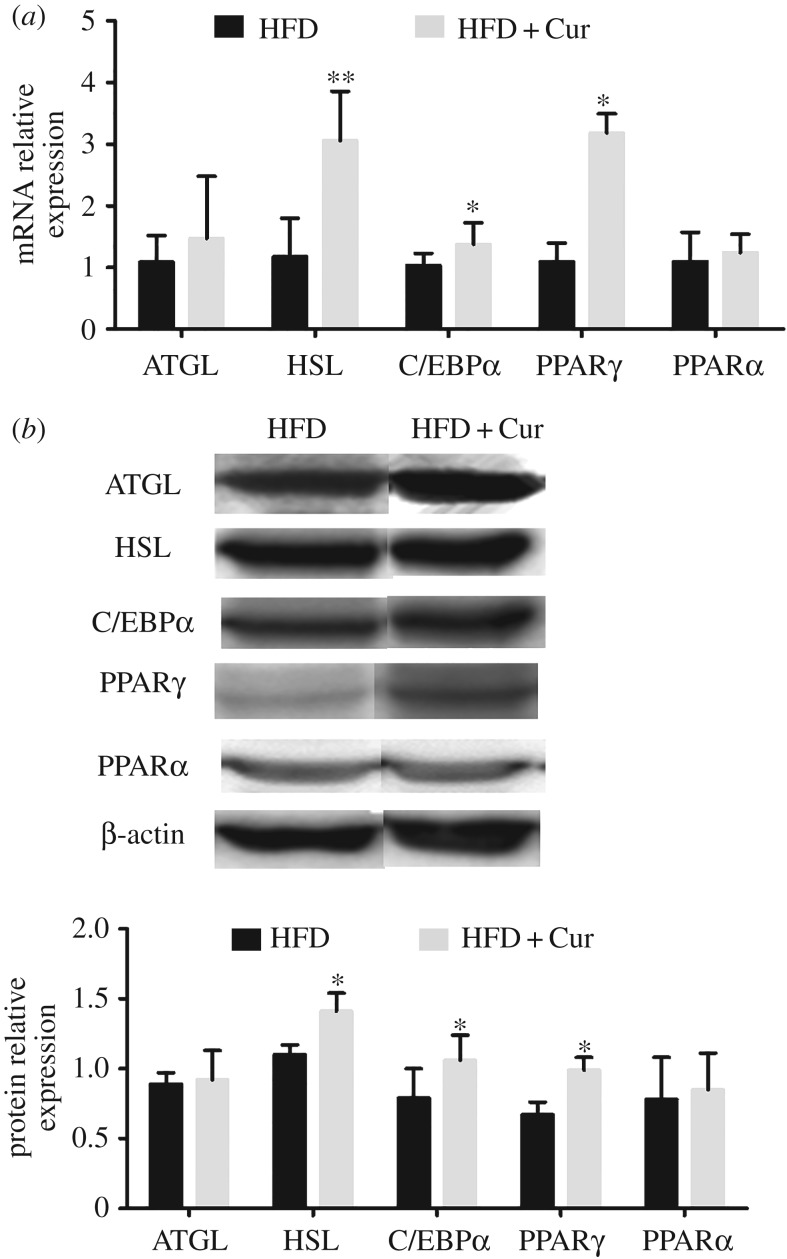
Curcumin increased the mRNA and protein expression in adipose tissue. (*a*) mRNA and (*b*) protein expression of ATGL, HSL, C/EBPα, PPARγ/α. Data are presented as mean ± s.e.m. in the HFD and HFD + Cur groups. Significant difference: *p < 0.05, **p < 0.01 versus the HFD group.

As shown in figure 5, curcumin upregulated the mRNA expression of ATGL, HSL, C/EBPα, PPARγ and PPARα by 1.4-, 2.6-, 1.3-, 2.9- and 1.1-fold, respectively, compared with the HFD group (figure 5*a*). The protein expressions of ATGL, HSL, PPARγ, C/EBPα and PPARα were consistent with the mRNA expression level (figure 5*b*). And protein expression levels are consistent with mRNA expressions. These results indicated that curcumin stimulated lipolysis of adipose tissue through regulating the expression of ATGL and HSL. In addition, curcumin promoted glucose uptake and enhanced insulin sensitivity via upregulating the C/EBPα and PPARγ level.

### 3.5. Effect of curcumin on glycolipid metabolism in 3T3-L1 adipocytes

**Figure 6.**
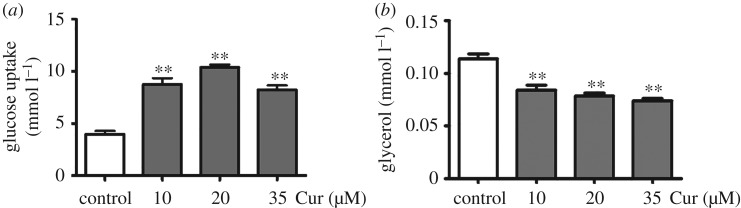
Effects of curcumin on glycolipid metabolism in 3T3-L1 adipocytes. (*a*) Glucose uptake and (*b*) glycerol release content in differentiated 3T3-L1 adipocytes. Data are presented as mean ± s.e.m. in each group. Significant difference: **p < 0.01 versus the control group (0 µM).

Curcumin increased basal glucose uptake of 3T3-L1 mature adipocytes by 1.2-, 1.6- and 1.0-fold in the presence of curcumin (10, 20 and 35 µM) compared with the control group (0 µM), and exhibited the maximum effect on glucose uptake at 20 µM (figure 6*a*). In addition, treatment with curcumin decreased the glycerol release by 74%, 69% and 64% compared with the control group (0 µM) (*p* < 0.01) (figure 6*b*).

### 3.6. Effect of curcumin on CCAAT/enhancer binding proteinα, peroxisome proliferator activated receptor γ and peroxisome proliferator activated receptor α expression in 3T3-L1 adipocytes

**Figure 7.**
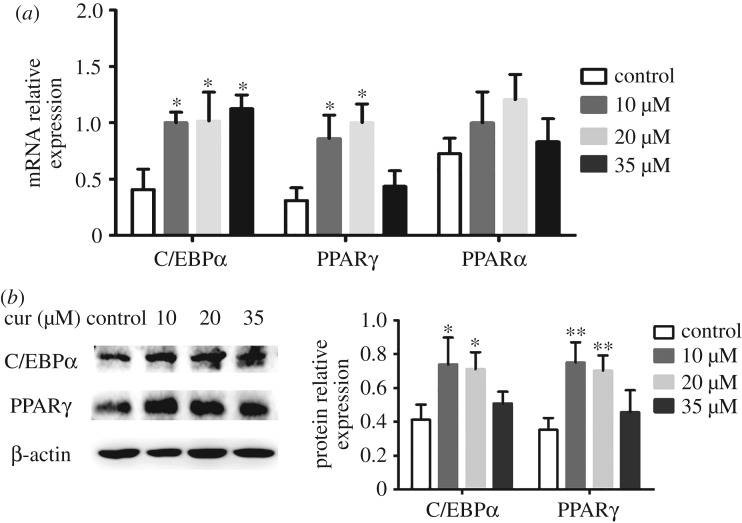
Effects of curcumin on the (*a*) mRNA expression of C/EBPα, PPARγ and PPARα and (*b*) protein expression of C/EBPα and PPARγ in 3T3-L1 adipocytes. Data are presented as mean ± s.e.m. in each group. Significant difference: *p < 0.05, **p < 0.01 versus the control group (0 µM).

The various doses of curcumin increased the mRNA expression of C/EBPα, PPARγ and PPARα in 3T3-L1 cells (figure 7*a*). The expressions of C/EBPα increased by 1.4-, 1.5- and 1.6-fold in the presence of curcumin (10, 20 and 35 µM) compared with the control group (*p* < 0.05). Similarly, expressions of PPARγ were also enhanced by 1.8- and 2.2-fold, respectively, at doses of 10 and 20 µM (*p* < 0.05). However, the mRNA expressions of PPARγ did not exhibit a significant difference at a dose of 35 µM (*p* > 0.05). Moreover, different dosages (10, 20 and 35 µM) of curcumin did not exert an effect on mRNA expression of PPARα compared with the control group in the current study (*p* > 0.05). Then, we verified the protein expression of C/EBPα and PPARγ in western blot analysis. The results showed that the protein expression of C/EBPα and PPARγ was enhanced by the treatment with curcumin, which was consistent with the results of mRNA expression (figure 7*b*). These results indicated that curcumin promoted glycolipid metabolism, which coincided with the *in vivo* experiments.

## 4. Discussion

In this present study, we investigated the effect of curcumin on anti-obesity and glycolipid metabolism and then explored the underlying mechanisms. Our major findings were that curcumin significantly reduced body weight, fat mass, blood serum lipid profiles and FBG level, and improved insulin resistance caused by obesity, indicating that curcumin acts on regulating glycolipid metabolism in HFD-induced obese C57BL/6 J mice. In differentiated 3T3-L1 adipocytes, curcumin increased glucose uptake and decreased glycerol release which coincided with the *in vivo* experiments.

Curcumin is a hydrophobic polyphenol extracted from turmeric, which has been the subject of intensive researches due to its wide pharmacological activities [18]. A few clinical trials have shown the therapeutic effects of curcumin on metabolic syndrome [19,20]. Moreover, Zhou *et al*. [21] showed that curcumin stimulates gene expression of PPARγ in activated hepatic stellate cells and interrupts PDGF and EGF signalling. In this study, we found that curcumin prevented body weight gain and reduced fat mass and exerted an anti-obesity effect in HFD-induced obese mice. In addition, insulin resistance is a widespread phenomenon in obesity and also a vital factor in the development of hypertension and hyperlipidaemia [22]. Moreover, in the few animal experiments that have been conducted, oral administration of curcumin significantly improved insulin resistance in STZ-induced diabetic rats and mice [23,24]. This study demonstrated that curcumin not only exerted an anti-obesity effect but also reduced FBG level and improved insulin sensitivity in HFD-induced obese mice. Besides, curcumin markedly promoted glucose uptake in differentiated 3T3-L1 adipocytes, consistent with the *in vivo* experiments. The above results indicated curcumin has the ability to treat and prevent obesity and diabetes mellitus.

Lipid metabolic disorders stimulate the excessive production of FFA in obese individuals, which subsequently decreases insulin-stimulated glucose uptake in the whole body [25,26]. Asai *et al*. [27] reported that curcumin lowered the level of FFA through stimulating the expression of ACO, and further improved lipid metabolism. In our study, we found curcumin markedly reduced the FFA level after curcumin administration in the HFD + Cur group. And in the *in vitro* experiment, curcumin decreased glycerol release in differentiated 3T3-L1 adipocytes. These results suggested that curcumin has a certain effect on regulating lipid metabolism dysfunction.

Adipose tissue functions as an endocrine organ secreting several cytokines and adipokines that are key determinants of whole-body energy homeostasis [6]. It is well known that PPARγ and C/EBPα mutually induce adipocyte differentiation and function [28]. Recently, genome-wide profiling revealed PPARγ and C/EBPα to have a high overlap in adipocytes, suggesting that cooperativeness could be mediated through common binding sites. Moreover, PPARγ and C/EBPα play an important role in driving insulin-stimulated glucose uptake [29]. In this study, curcumin increased the mRNA and protein expression of PPARγ and C/EBPα in mice and in differentiated 3T3-L1 cells. These results indicated that curcumin promoted glucose uptake and improved insulin resistance through upregulating the expression of PPARγ and C/EBPα. But high dose (35 µM) of curcumin did not significantly upregulate the protein expression of PPARγ and C/EBPα in 3T3-L1 cells. We hypothesize that 35 µM curcumin may have an effect on the activity of some enzymes in the process of translating into proteins, so further study and discussions deserve to be explored in the future.

In addition, we found that curcumin administration led to the activation of lipolysis in WAT. WAT is a metabolic flexible tissue, which is important for energy storage or energy source release in the form of adipogenesis and lipolysis of TGs [5]. As reported, activation of PPARγ enhanced lipolysis of circulating TGs and their storage in adipose tissue [30,31]. A study reported that PPARγ signalling regulated the lipolytic response through its modulatory effect on adipose tissue lipases including ATGL and HSL that hydrolysed intracellular triacylglycerol and diacylglycerol to release FFA and glycerol [32]. In this study, we found that curcumin significantly increased the mRNA and protein expression of ATGL and HSL in HFD-induced obese mice. All above results indicate that curcumin improved glycolipid metabolism and enhanced insulin sensitivity through regulating PPARγ signalling pathway *in vivo* and *in vitro*.

## 5. Conclusion

Curcumin administration reduced body weight, lowered the blood glucose level and serum lipid profiles and improved insulin sensitivity in obese mice through regulating the PPARγ signalling in adipose tissue. The results suggest that curcumin seems to offer potency as a traditional plant extract that is useful to prevent and treat obesity and diabetes.

## Supplementary Material

Data S1

## Supplementary Material

Data S2

## Supplementary Material

Data S3

## Supplementary Material

Data S4

## Supplementary Material

Data S5

## Data accessibility

All relevant data are within the paper and the electronic supplementary material files.
